# Identification of Plant DNA in Adults of the Phytoplasma Vector *Cacopsylla picta* Helps Understanding Its Feeding Behavior

**DOI:** 10.3390/insects11120835

**Published:** 2020-11-26

**Authors:** Dana Barthel, Hannes Schuler, Jonas Galli, Luigimaria Borruso, Jacob Geier, Katrin Heer, Daniel Burckhardt, Katrin Janik

**Affiliations:** 1 Laimburg Research Centre, Laimburg 6, Pfatten (Vadena), IT-39040 Auer (Ora), Italy; 2 Faculty of Science and Technology, Free University of Bozen-Bolzano, IT-39100 Bozen (Bolzano), Italy; hannes.schuler@unibz.it (H.S.); luigimaria.borruso@unibz.it (L.B.); 3 Competence Centre Plant Health, Free University of Bozen-Bolzano, IT-39100 Bozen (Bolzano), Italy; 4 Department of Forest and Soil Sciences, BOKU, University of Natural Resources and Life Sciences Vienna, A-1190 Vienna, Austria; jonasgalli95@gmail.com; 5 Department of Botany, Leopold-Franzens-Universität Innsbruck, Sternwartestraße 15, A-6020 Innsbruck, Austria; jacob.geier@laimburg.it; 6 Faculty of Biology—Conservation Biology, Philipps Universität Marburg, Karl-von-Frisch-Straße 8, D-35043 Marburg, Germany; katrin.heer@uni-marburg.de; 7 Naturhistorisches Museum, Augustinergasse 2, CH-4001 Basel, Switzerland; Daniel.Burckhardt@bs.ch

**Keywords:** apple proliferation, feeding behavior, food plants, phloem feeding, Psylloidea, *rbcLa*, sequencing, *trnH*

## Abstract

**Simple Summary:**

*Cacopsylla picta* is an insect vector of apple proliferation phytoplasma, the causative bacterial agent of apple proliferation disease. In this study, we provide an answer to the open question of whether adult *Cacopsylla picta* feed from other plants than their known host, the apple plant. We collected *Cacopsylla picta* specimens from apple trees and analyzed the composition of plant DNA ingested by these insects. By applying a state-of-the art sequencing approach, we show, for the first time, that *Cacopsylla picta* feeds from a wide range of woody and herbaceous plant species. Our results are important for a better understanding of the biology and feeding behavior of *Cacopsylla picta*. Since this insect is an efficient vector of apple proliferation phytoplasma, our results are also important to define potential reservoir plants that might be involved in the transmissive cycle of this pathogen. This study thus provides important data of practical relevance.

**Abstract:**

Apple proliferation is an economically important disease and a threat for commercial apple cultivation. The causative pathogen, the bacterium ‘*Candidatus* Phytoplasma mali’, is mainly transmitted by *Cacopsylla picta*, a phloem-feeding insect that develops on the apple tree (*Malus* spp.). To investigate the feeding behavior of adults of the phytoplasma vector *Cacopsylla picta* in more detail, we used deep sequencing technology to identify plant-specific DNA ingested by the insect. Adult psyllids were collected in different apple orchards in the Trentino-South Tyrol region of northern Italy. DNA from the whole body of the insect was extracted and analyzed for the presence of plant DNA by performing PCR with two plant-specific primers that target the chloroplast regions *trnH-psbA* and *rbcLa*. DNA from 23 plant genera (*trnH*) and four plant families (*rbcLa*) of woody and herbaceous plant taxa was detected. Up to six and three plant genera and families, respectively, could be determined in single specimens. The results of this study contribute to a better understanding of the feeding behavior of adult *Cacopsylla picta*.

## 1. Introduction

Apple proliferation (AP) is an economically important disease in commercial apple cultivation and is caused by the cell wall-less bacterium ‘*Candidatus* Phytoplasma mali’ [1]. Formation of witches’ brooms and enlarged, dentate stipules are specific symptoms that unambiguously characterize this disease [2,3]. Infected plants produce small, tasteless and colorless fruits that are not marketable [3]. Since there is no curative treatment for the infected trees, they have to be uprooted to prevent further spread of the pathogen. The financial loss caused by the quality loss and financial expenditure of tree removal was about EUR 100 million in Italy in 2001 and about EUR 50 million in the province of South Tyrol in 2006 and 2013 [4,5]. AP is a constant threat in apple-growing regions. The pathogen is transmitted by the phloem-feeding insects *Cacopsylla picta* (Foerster 1848) (synonym *Psylla costalis*; Flor 1861) (Hemiptera: Sternorrhyncha: Psyllidae), *C. melanoneura* (Foerster 1848) and *Fieberiella florii* (Stål 1864) (Hemiptera: Auchenorrhyncha: Cicadellidae) [6,7,8,9,10,11]. ‘*Ca.* P. mali’ infects *Malus* spp., but the pathogen could be also detected in wild and ornamental plants (reviewed in Janik et al.; [5]). In plants, the bacterium colonizes the phloem, and *C. picta* can, thus, acquire the pathogen during feeding on an infected plant. In a competent vector, the bacterium replicates and migrates to the salivary glands and is released to the insect’s saliva. Due to saliva release during psyllid feeding activity, the pathogen can be further transmitted to new plants [12]. As *C. picta* can transmit ‘*Ca.* P. mali’ transovarially to its progeny, a single mother individual can produce hundreds of infected descendants and even a small *C. picta* population can lead to severe AP outbreaks [13]. *C. picta* is considered the main vector of the AP-inducing pathogen [14,15,16].

*Cacopsylla picta* is univoltine, i.e., it reproduces once a year. The insect is considered monophagous, meaning that the immatures feed and develop exclusively on one host plant taxon. The host plant taxon on which *C. picta* immatures feed and develop is *Malus* spp. [17,18]. In northern Italy, *C. picta*’s reproduction takes place from mid-March until the beginning of August [19,20]. During this period, the overwintered adults—the so-called remigrants—of *C. picta* can be detected in apple orchards until the beginning of June, while the young adults of the new generation—the so-called emigrants—occur from the end of June until the beginning of August [19,20]. During the rest of the year, adults of *C. picta* dwell on shelter plants, mostly conifers at higher elevations [21]. Immature psyllids are generally restricted to one or several closely related plant species on which they can complete their development. The more mobile adults are often found on various plants on which they presumably feed (= food plants) or overwinter (= shelter plants) [22]. To overcome long distances between host and shelter plants, it is assumed that the insects use convection winds and are passively dispersed by aerial currents [21]. In this manner, psyllids can be transferred by up to as much as 27 km [23]. While the host plant of *C. picta* is known, it has been suggested, but not proven, that adult psyllids also feed on other plant taxa [17]. Detailed literature about the feeding behavior of adult *C. picta* is missing. Only observations regarding the presence of adult *C. picta* on diverse plant species have been reported so far [17,18,24], but it has not been proven that the psyllids feed on these plants.

Psyllids subsist on nutrients, such as minerals, amino acids and sugars, which are the most abundant components of phloem sap [25,26]. With their two mandibular and two maxillary stylets, the insects penetrate the plant tissue to reach the phloem sap in the sieve tubes [27,28,29,30]. Some plant species prevent insect feeding by applying diverse defense strategies, such as the release of inhibitory or toxic compounds [31] or the occlusion or constriction of sieve pores with callose or phloem proteins [32,33,34,35]. Some plants possess a thick-walled sclerenchyma around the phloem which functions as a protective layer against insect feeding [30,36]. Due to successful defense strategies of the plant, psyllids may probe (= stylet penetration) without actually being able to feed on the plant. Plants which do not serve as feeding plants are called casual plants [22]. Even though insertion of the stylet is a time-consuming process for the insect and can take longer than the actual feeding itself [27,28], probing is indispensable for the psyllid to identify its host plant [37,38].

To explore the diet composition of herbivorous insects, different methods have been established. Field observations are time consuming and often error-prone, mainly because this method does not allow the determination of whether the plant from which the insect was collected was a casual, feeding or a shelter plant [39]. For plant-chewing insects, a microscopical gut content analysis can give unambiguous insights into feeding behavior. This analysis allows to determine post-prandial residues of plants based on plant morphological characteristics [40,41]. However, since psyllids only ingest phloem sap, no morphologically determinable plant structures are available for a gut content analysis. Detection and identification of plant species-specific DNA regions in the insect does not require morphological plant identification [39,42] and this molecular identification has just recently been shown to be an appropriate method to detect plant DNA in phloem-feeding insects [43,44]. Even though phloem cells lack the capacity to synthesize nucleic acids [31], the phloem sap presumably contains plant DNA, e.g., deriving from cellular debris of the phloem tissue used for sieve pore sealing [45]. Phloem companion and mesophyll cells contain chloroplasts and, thus, it can be assumed that chloroplast-derived DNA is also present in the phloem sap [46]. Furthermore, during the stylet penetration process, plant cells are taken up by the insect as well [43]. However, identification of plant DNA in the phloem-feeding insect does not indicate if the insect fed or only probed the plant tissue, or in other words, if the plant DNA derives from a feeding or a casual plant [22].

Knowledge about the dietary composition of phytoplasma vectors is important to determine the range of plants approached by the insect by feeding or probing [12]. An improved understanding of the feeding habits of *C. picta* and its dispersal provides the basis for the identification of so-far unknown reservoir plants involved in phytoplasma transmission. We applied highly sensitive deep sequencing of chloroplast amplicons to detect plant DNA in whole-body extracts from adult *C. picta* and gained insights into the feeding behavior of this important phytoplasma vector.

## 2. Materials and Methods

### 2.1. Insect Sampling and Morphological and Molecular Species Identification

From 2012 to 2016, ninety-five adult *Cacopsylla picta* individuals were collected directly from apple trees. Insects were captured with a conical tunnel-like beat tray connected to a collection vessel or with yellow sticky traps that were placed on apple trees. Sampling was carried out in 14 different apple orchards in the Trentino-South Tyrol (Trentino-Alto Adige) region of northern Italy (Table 1). Dietary composition of the insects before the catch was unknown. Remigrant specimens were collected in March, April and May from all 14 orchards; emigrant specimens were collected in June and July in one orchard (Mais). The captured insects were morphologically determined [18]. DNA of each single individual was extracted using the DNeasy^®^ Blood and Tissue Kit (Qiagen, Hilden, Germany) according to the manufacturer’s instruction. DNA was eluted with 100 µL Tris-EDTA elution buffer (Tris-Cl 10 mM; EDTA 0.5 mM; pH 9.0) (AppliChem GmbH, Darmstadt, Germany). Species confirmation was performed using restriction fragment length polymorphism according to Oettl and Schlink [47]. Briefly, PCR was performed in a final volume of 20 µL containing 1× Colorless GoTaq^®^ Buffer, 0.2 mM dNTPs, 0.7 mM of each primer and 0.02 U GoTaq^®^ DNA polymerase (Promega, Madison, USA) under the following conditions: 2 min at 95 °C; 45 cycles at 95 °C for 30 s, 46 °C for 30 s and 72 °C for 1 min; and 5 min at 72 °C. Restriction was performed with 10 µL of the amplicon and 0.5 U TaqαI restriction enzyme in 1× CutSmart^®^ (New England Biolabs Inc., Ipswich, USA) buffer at 65 °C for 4 h. DNA fragments were separated using 2.0% MetaPhor™ agarose (Lonza, Basel, Switzerland) gels and stained with ethidium bromide. Only ‘*Ca.* P. mali’ non-infected *C. picta* specimens were used in this study. The infection status was determined as described in Mittelberger and co-authors [13].

### 2.2. Molecular Analysis and Deep Sequencing

Plant DNA was amplified using the primer pairs psbA-F and trnH-R [48,49] as well as rbcLa-F and rbcLa-R [50,51]. Reaction mixtures of 10 µL contained 1 µL of template DNA (ca. 50 ng), 2 µL of 5× MyTaq™ Buffer, 0.75 µL of each primer (10 µM), 1 µL of BioStab PCR optimizer (II) (Merck, Darmstadt, Germany), 0.15 µL of MyTaq™ Polymerase (5 U/µl) (Meridian Bioscience, London, UK) and 4.35 µL molecular grade water. PCR and amplicon sequencing were conducted at LGC Genomics GmbH (Berlin, Germany). The PCR was performed as follows: Initial denaturation for 2 min at 95 °C followed by 35 cycles of 15 s denaturation at 96 °C, 30 s annealing at 58 °C and 90 s extension at 68 °C; final elongation was performed for 1 min at 72 °C. Illumina sequencing adapters were attached to the purified PCR products. An extended sequence read length of up to 2 × 300 base pairs (bp) obtaining paired-end sequencing was performed on an Illumina^®^ MiSeq^®^ lane (Illumina, Inc., San Diego, CA, USA).

### 2.3. Data Analysis

Amplicon sizes were unexpectedly long for some plant species and forward and reverse sequence reads could not be unambiguously assembled. Thus, for the data analysis, only the forward sequence reads were used. A quality control of the raw sequence reads was done in R with package DADA2 [52]; sequence reads shorter than 200 bp or with a Phred score of less than 2 were discarded. The operational taxonomic unit (OTU) assignment was performed with DADA2 and a further clustering of the OTU sequences was performed with the tool cd-hit with a sequence identity of 99%. The OTU sequence with the most reads was taken as the reference sequence for each cluster. In order to combine the clustering results with the read counts, the data were read in Eclipse using Java and the read counts were summed up for every cluster. Clusters with a minimum of 100 reads were retained and all others discarded. The taxonomical identification was done with an open blast on the National Center for Biotechnology Information database (NCBI) (Bethesda, MD, USA), in which each OTU was individually matched against the complete nucleotide database.

Only BLAST hits with a query cover of at least 99% were considered to determ OTUs. The OTUs generated with the *trnH* locus sequences allowed an assignment up to the taxonomic genus-level, and the results of the *rbcLa* locus sequencing, up to the taxonomic family-level. OTUs of the same genus (*trnH*) or of the same family (*rbcLa*) were combined. To verify the validity of the OTUs, the taxa were matched to a list of plants naturally occurring in the study region [53,54]. Insects from which no reads passed the sequence quality filtering were excluded.

For both loci, a Mann–Whitney U test was performed to compare the numbers of reads and the number of different plant taxa detected in specimens collected with the beat tray or the sticky traps. All analyses were carried out in the R statistical software (v. 3.1.3) (R studio, Boston, MA, USA).

### 2.4. Longevity under Starvation

Since a reference value for the longevity of *C. picta* under starvation was missing, a survival experiment was carried out. Fifteen emigrants of *C. picta* which derived from a rearing experiment were transferred to an empty 50-mL polypropylene test tube and kept without a food source. A control set built of another fifteen emigrants of *C. picta* was transferred to another test tube that was put over a branch of a potted spruce and was tightly closed with gauze. All insects were kept in a rearing chamber with 75% relative humidity, 21 °C and a day length of 19 h. The number of alive or dead insects was counted every day. The experiment ended after 10 days.

### 2.5. Pollen Staining

As deep sequencing approaches are sensitive to contamination, we investigated the effect of airborne pollen contamination in five *C. picta* specimens, which were caught with a beating tray in the sampling location Tenna/Bosentino in May 2015. The insects were examined with a light microscope. The staining procedure was performed under a laminar flow to prevent contamination by airborne pollen. The insect was placed on a microscope slide and covered with a few drops of soapsuds (ca. 10 mL water with 3 drops of curd soap). By carefully shaking the insect on the slide, the hydrophobic pollen grains were detached from the insect’s body and dispersed in the soapsuds. The sample was air-dried until the soapsuds were completely evaporated. The sample was then covered with a drop of the dye/embedding solution (containing 0.5 mL fuchsin-ethanol solution (0.2%), 15 g polyvinyl alcohol (embedding agent, fully hydrolyzed) and 50 mL glycerin in 150 mL water) and covered with a cover slip. The sample was incubated for 24 h at 22 °C (room temperature). All pollen grains dispersed in the detergent and attached to the insect’s body were counted.

## 3. Results

**Figure 1:**
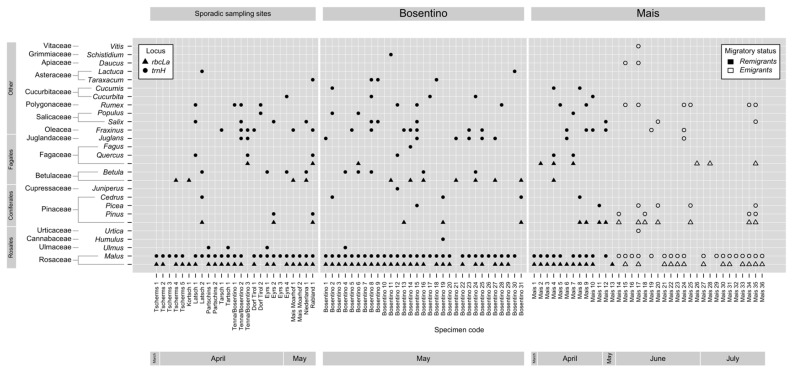
Dietary composition for each *C. picta* specimen. A total of 95 *C. picta* individuals were sequenced. In three of these specimens, no plant DNA was found and they were excluded from further analysis. The 92 individuals in which plant DNA was detectable are shown in the graph. The graph is divided into three sections regarding the main sample sites “Bosentino” and “Mais” and “sporadic sampling sites”. Remigrants are represented by black symbols and emigrants by white symbols. Triangles (p) and circles (l) indicate sequencing results generated with the *rbcLa*- or *trnH*-specific primers, respectively. Quantitative information for every specimen can be found in supplementary Tables S1 and S2.

After a stringent quality control, 393,442 (*trnH*) and 91,806 (*rbcLa*) sequence reads were analyzed. On average, 4141 and 144 sequence reads per insect were recorded (*n* = 92) for *trnH* and *rbcLa*, respectively (Table A1). One remigrant from Bosentino and one from Mais and one emigrant from Mais were excluded because no plant DNA could be detected using the *trnH* or the *rbcLa* primers. Sequencing of the *rbcLa* amplicon revealed five OTUs that could not be unambiguously assigned to a particular genus but only to a plant family. Considering only plants that naturally occur in the study region, the sequence of the OTU that was assigned to Rosaceae might derive from the genera *Malus*, *Pyrus*, *Sorbus*, *Crataegus* or *Amelanchier*. The *rbcLa* sequences of these five genera are indistinguishable and were, thus, designated the Rosaceae OTU. In 75% of the *C. picta* individuals, Rosaceae-specific *rbcLa* sequences were found. Three other OTUs were identified. One OTU could be assigned to the genera *Pinus* or *Picea* of the Pinaceae family and another OTU was ambiguously assigned to *Cedrus* or *Larix* of the Pinaceae family. Thus, these two OTUs were allocated to the Pinaceae family. Pinaceae DNA was verified in almost every fifth *C. picta*. DNA of Betulaceae, which indistinguishably match to *Betula*, *Ostrya* or *Carpinus*, was detected in 13% of the *C. picta* individuals. Sequences from Fagaceae could derive from *Quercus* or *Castanea* and were found in 10% of the specimens (Table 2 and Figure 1). Taken together, *rbcLa* sequence assignment allowed to identify DNA derived from four families of woody plants in *C. picta* (Table 2 and Figure 1).

The sequence analysis of the *trnH* amplicon allowed a taxonomic determination up to the genus level, taking into account only plants that naturally occur in the study region. In total, DNA of 23 plant genera comprising 12 woody and 11 herbaceous plant taxa was identified (Table 2 and Figure 1). Specific *trnH* sequences from *Malus* were detected in 87% of the specimens. In about every fifth insect, *Fraxinus* (Oleaceae)-specific *trnH* DNA was found. *Rumex* (Polygonaceae) were found in 16% of the specimens. DNA from *Salix* (Salicaceae), *Juglans* (Juglandaceae) and *Betula* (Betulaceae) was detected in 11% and 10% of the insects, respectively. *Pinus-* and *Picea* (both Pinaceae)-specific DNA was found in 7% of the analyzed *C. picta* individuals. DNA deriving from cultivated plants, e.g., *Cucurbita*, *Cucumis* (both Cucurbitaceae) and *Vitis* (Vitaceae), was verified in 5% or less of the specimens. The genera *Daucus* (Apiaceae), *Taraxacum* and *Lactuca* (both Asteraceae), *Humulus* (Cannabaceae), *Juniperus* (Cupressaceae), *Populus* (Salicaceae), *Ulmus* (Ulmaceae), *Urtica* (Urticaceae) and *Schistidium* (Grimmiaceae) were identified in ≤5% of the *C. picta* individuals (Table 2 and Figure 1).

The mean number of sequence reads per insect and taxa was less than 100 for the *rbcLa* locus, while the mean number of reads per taxa varied between 3842 (*Malus*) and 26 (*Quercus*) counts for the *trnH* locus (Table 2, Tables S1 and S2). The number of reads (both loci) from insects that were collected with the beating tray did not significantly differ from those that were collected with sticky traps (Figure A2a).

**Figure 2:**
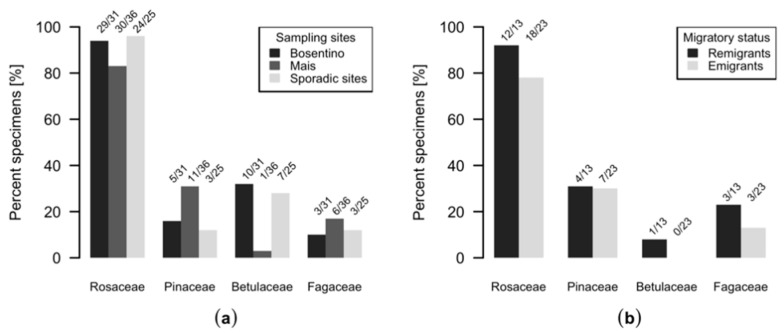
Detection of selected plant families (*trnH* and *rbcLa*) (**a**) in insects deriving from different sampling locations and (**b**) in remigrants and emigrants deriving from Mais.

On average, one plant family and two genera were identified per insect and a maximum of three plant families and six genera in a single individual could be identified (Figure 1). Regardless of the analyzed locus, we could not observe differences regarding the number of different plant taxa in specimens collected with the beat tray or sticky traps (Figure A2b). Most *C. picta* individuals, i.e., 90%, were positive for Rosaceae (*rbcLa*) or *Malus* (*trnH*) at 72% for both (Figure 1). Figure 2 bundles the information from Figure 1 regarding the presence of Rosaceae, Pinaceae, Betulaceae and Fagaceae DNA in insects from different sampling sites. DNA from Betulaceae was detected in 32% of the insects from Bosentino, 3% of the insects from Mais and 28% of the specimens from other orchards (Figure 2). DNA from Fagaceae was found in 10% of the individuals from Bosentino, 17% of the *C. picta* individuals from Mais and 14% of those from other orchards (Figure 2). Pinaceae DNA was found in 16% of individuals from Bosentino, 31% of those from Mais and 14% of those from other orchards (Figure 1 and Figure 2). In Mais, remigrants and emigrants were sampled and 69% of the remigrants of *C. picta* carried DNA from taxa other than Rosaceae or Pinaceae. DNA from these two families was detected in only 43% of the emigrants (Figure 1 and Figure 2).

**Figure A1:**
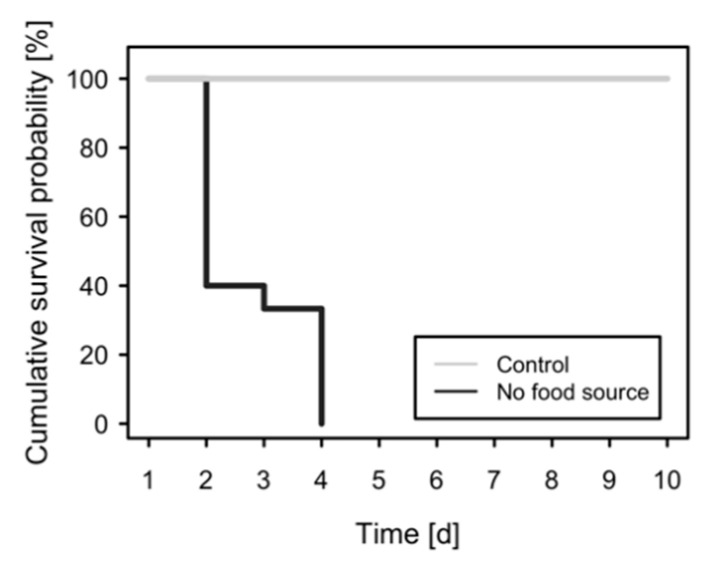
Mortality of reared *C. picta* emigrants, kept without food and water.

In the test tubes without a food source, all emigrants of *C. picta* died within four days (Figure A1) and the mean longevity under starvation was 1.6 d. All specimens that had the possibility to feed on spruce survived for the entire experiment duration of 10 days.

**Figure A2:**
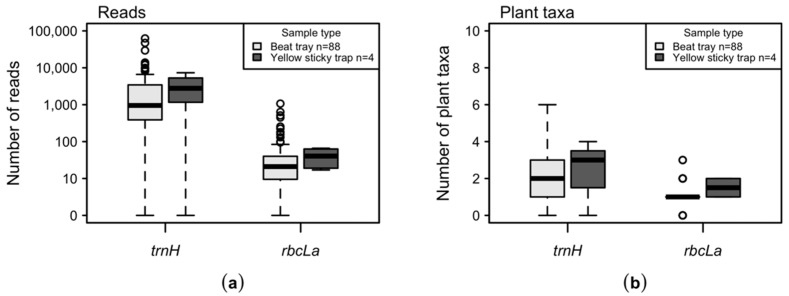
Comparison of sample type: (**a**) the numbers of reads displayed on a logarithmic scale according to the sample type and (**b**) the numbers of plant taxa in specimens according to the sample type.

Pollen was found in very low numbers on the insects with a maximum of four pollen grains per specimen (Table A1, Figure A2a,b).

## 4. Discussion

The host plant of *C. picta* is known and it has been suggested [17], but not proven, that adult psyllids also feed on other plant taxa. *C. picta* can survive for up to four days without food (Figure A2), but the insect lives for only four or five months a year on its host [19,20]. This leads to the conclusion that *C. picta* must feed from plants other than *Malus*. Detailed literature about the feeding behavior of adult *C. picta* is missing. Observations regarding the presence of adult *C. picta* on diverse plant species have been reported [17,18,24], but it remains unclear if the insects feed on these plants. Therefore, we applied an amplicon deep sequencing approach to identify plant DNA in adult *C. picta* to shed light on whether adult *C. picta* feed on plants other than the known host plant, *Malus* spp.

An unexpected diversity of DNA from different non-host plants—woody, herbaceous and different crop plants—was detected in adult *C. picta* specimens (Table 1 and Figure 1). In a study from Ullman and McLean [55], it was shown that *Cacopsylla pyricola* feeds on peach, which is a plant that does not serve as a host plant for the nymphal development of this psyllid. Similar to our results, a broad range of non-host plant DNA was detected in adults of following North American psyllids: *Aphalara loca* (Caldwell 1937) (Hemiptera: Sternorrhyncha: Aphalaridae), *Bactericera cockerelli* (Šulc 1909) (Hemiptera: Sternorrhyncha: Triozidae), *Cacopsylla pyricola* (Foerster 1848), *Diaphorina citri* (Kuwayama 1908) (Hemiptera: Sternorrhyncha: Liviidae) and willow psyllids [44]. In *C. pyricola*, for example, *Salix*, *Juniperus*, *Pinus*, *Humulus* and melon (Cucurbitaceae) were identified [44]. These plant taxa have also been determined in *C. picta* in our study. Furthermore, DNA from Rosaceae (*rbcLa*), Betulaceae (*rbcLa*), Fagaceae (*rbcLa*) and Quercus (*trnH*) was found in *C. picta*. *Cacopsylla picta* transmits the plant pathogen ‘*Ca.* P. mali’, the causative agent of apple proliferation disease. This pathogen was also found in other plant species, e.g., wild, ornamental and cultivated plants belonging to Betulaceae, Fagaceae and Rosaceae: *Carpinus betulus* [56], *Corylus avellana* [57], *Prunus salicina*, *Pyrus communis*, *Pyrus pyrifolia* [58], *Prunus avium*, *Quercus robur* and *Quercus rubra* [3]. This coincidence, i.e., that *C. picta* transmits the phytoplasma during feeding and that the phytoplasma can be found in the above-mentioned plants, supports the assumption that adults of *C. picta* exploit a wide range of feeding plants. However, the role of these plants as reservoir plants in the transmissive cycle of ‘*Ca.* P. mali’ is unclear. Generally, every feeding plant of *C. picta* might play a role as a reservoir plant, and thus, our results provide an important starting point to elucidate which plant taxa are worth being considered as potential reservoir plants. However, it remains unknown if acquisition and transmission is feasible when psyllids feed from non-host plants. A pre-requisition that a plant plays a role as a reservoir plant is that the phytoplasma can colonize its phloem and that it can be transmitted to the insect. Since the dwelling time of the insect on its host plant is presumably longer than on its feeding or casual plants, the efficiency of pathogen acquisition from an infected insect host plant is probably higher. Similarly, it can be assumed that the long dwelling time increases the probability that an infected insect transmits the pathogen to its host plant. If a pathogen can colonize the plant but cannot be transmitted to the vector insect during feeding, the plant is a dead-end host [12]. The role of feeding plants of adult *C. picta* in the ‘*Ca.* P. mali’ transmission cycle has not been analyzed, yet.

The detection of DNA from a high number of non-host plants in adults of *C. picta* emphasizes that these insects do not stay exclusively on *Malus* spp. during their dwelling time in the apple orchard. The insects might have been transported to the scrub layer or to plants in the vicinity of the apple orchard passively by wind or rain, or they actively dispersed in the surrounding area, e.g., as a reaction to high population pressure [22,59]. *C. picta* remigrants are sexually mature and search for mating partners and oviposition sites on *Malus* spp. [16,60]. Until now, the food plant range of *C. picta* remigrants has been scarcely described in the literature. Forno and colleagues observed that emigrants of *C. picta* are present in the scrub layer during their presence in the apple orchard [24]. It needs to be considered that emigrants have a different motivation than remigrants to leave their host plant. Emigrants of *C. melanoneura* tend to disperse from the host and Mayer and colleagues observed that they develop this impulse already two days after hatching [61]. Further, Ossiannilsson, as well as Lauterer, described that emigrants of *C. picta* move to annual plants of the genera *Brassica*, *Mentha*, *Vicia*, *Phaseolus*, *Pisum* or *Avena* and later to shelter plants [18,60]. The observations of these authors and the results of our study point out that herbaceous plants, which *C. picta* has rarely been associated with, also play a certain role for transit and feeding in the life cycle of this insect.

It is known that *C. picta* overwinters on conifers (Coniferales) [17,21], but it is still under debate if they feed on shelter plants. Gallinger and Gross demonstrated with the use of electronic penetration graphs that *Cacopsylla pruni* (Scopoli 1763) feeds on conifers under laboratory conditions [62]. The results of our study show, for the first time, that DNA from *Picea*, *Pinus* and *Cedrus* can be detected in *C. picta*. DNA from Pinaceae was also found in *C. pyricola* [44]. These findings strengthen the hypothesis that the members of the genus *Cacopsylla* feed on plants of the Pinaceae family.

Interestingly, Pinaceae DNA was found in remigrants and emigrants in almost equal proportions. It can be assumed that *C. picta* adults also probed or fed on conifers on their way to the apple orchard or in the surrounding vegetation. All emigrants were caught in the Mais orchard, which is located on a mountainside at around 700 m above sea level. Thus, it is possible that emigrants coming from the lower valleys passed this orchard to reach higher mountain regions, where their coniferous winter shelter plants are located. In one of the emigrant specimens, *Vitis* DNA was identified, but the nearest vineyard is located about 500 m from the sampling location. This supports the hypothesis that some of the emigrants arrived from apple orchards in the lower located valley. Furthermore, our data indicate that *C. picta* emigrants already begin to migrate to shelter plants during summer, as it was also observed for *C. melanoneura* [61,63]. This suggests that adult *C. picta* feed on different plants during migration.

DNA from Rosaceae was found in almost all analyzed individuals and with the taxonomically-deeper-reaching *trnH* sequencing approach, *Malus* was identified as the only member of the Rosaceae family present in the analyzed insects (Figure 1 and Figure 2). As expected, most reads could be assigned to *Malus*, the host on which *C. picta* feeds and develops (Table 1). The presence of DNA of the known host plant in the phloem-feeding insect indicates that the applied sequencing approach is applicable for the detection of plant DNA in *C. picta*. No other plant genus was found as abundantly in the insects as *Malus*. To determine whether a particular psyllid used other plants for feeding or probing purposes, the amount of plant DNA found in the insect might be indicative. The abundance of sequence reads strongly depends on how much plant material the insect ingested and how much time passed since the feeding or probing event [44]. During the digestive process, plant DNA may be partially or completely degraded [64]. The read numbers may provide a rough idea of how much plant DNA was ingested, but a detailed interpretation of the read numbers is not recommended [44]. Based on these limitations and the fact that significant amounts of DNA might be ingested via sources other than the phloem sap (e.g., during cellular penetration), we cannot define whether the identified plants serve as feeding or as casual plants. The two collection methods. i.e., beat tray vs. sticky trap, did not differ regarding the number of sequence reads or the number of different taxa that were detected. This indicates that chemical reagents on the sticky trap and the circumstance that the insect has been probably dead since days did not technically hamper DNA extraction and quality. This is in line with the findings described by Cooper and co-authors [43].

We found that the dietary composition of *C. picta* is influenced by the fauna at the sampling location. Betulaceae DNA was mostly identified in specimens from Bosentino, while Pinaceae DNA was identified in specimens from Mais (Figure 2). Interestingly, *Fraxinus* DNA was found in about 20% of the insects from seven different sampling sites. Thus, it is possible that *C. picta* is particularly attracted to this plant genus.

It is unknown how long ingested plant DNA can be detected in *C. picta*. In the cell rupture feeding bug *Apolygus lucorum* (Meyer-Dür 1843) (Hemiptera: Heteroptera: Miridae) [65,66], plant DNA was amplifiable up to 20 h after the feeding event [67]. In *Drosophila suzukii* (Matsumura 1931) (Diptera: Brachycera: Drosophilidae), a sponging-feeding insect, plant DNA was detectable for a maximum of 48 h [64]. However, due to its different feeding behavior, it can be assumed that *Drosophila* ingests more plant DNA than a phloem-feeding insect. In a study with the phloem-feeding *B. cockerelli,* it could be shown in a deep sequencing approach that plant DNA remains detectable for an extended period of at least two weeks after the feeding plant switch [68]. Considering that we found a broad range of plant DNA from different genera in *C. picta,* it can be assumed that these psyllids also feed on or probe other plants in the surrounding of the host plant, even during their presence in the apple orchard. Psyllids are short-term flyers or they move by jumping; additionally, their movement is undirected and their activity is temperature-dependent [69,70,71,72]. Thus, it is unlikely that psyllids can actively overcome large distances, but they can be passively transported over longer distances by wind [21,22,23]. Considering the mode of translocation and the fact that DNA in a phloem-feeder can be detected for a prolonged time [68], it can be assumed that plants that are far from the sampling site can be detected by our method.

A surface decontamination of the insects was not performed because it cannot be ruled out that DNA destructing agents commonly used for decontamination penetrate the insect’s surface and destroy the minimal amounts of plant DNA present in the phloem-feeding insects’ intestinal system. On the other hand, a short decontamination time or low concentrations of decontamination solutions do not assure that the actual contaminant is removed. The most uncontrollable source of contamination is pollen, which can be air- or plant-borne. Pollen might firmly stick to the insect’s body and, thus, be a source of contamination since it is not easily removed during the preparation steps. Furthermore, whether an insect carries pollen contamination cannot be evaluated by the naked eye. The doubt of detecting contamination-derived amplicons was dispelled because we only found minimal amounts of pollen grains and did not observe any other plant-derived particles on the insect’s body (Table A2 and Figure A1). To further reduce the probability that plant DNA derived from surface-attached pollen is amplified, primers were used that target chloroplastic DNA. Pollen contains these organelles only in minor and negligible quantities [73,74,75]. During the quality processing of the sequence results, sequences with abundances less than 100 reads were not considered. Thus, considering the fact that only minimal amounts of pollen were found on the insects’ bodies, in combination with the use of primers that amplify chloroplast loci and the stringent quality control, the probability to generate artifact sequencing results from surface-attached particles was negligible. Beside this, it could be shown in a previous study, which was conducted with psyllids deriving from a colony, that decontamination did not improve the overall sequencing results [43].

In summary, our data show, for the first time, that adult *C. picta* harbor DNA from many different plant taxa, proving that adult *C. picta* are feeding and probing on several different plant species. We emphasize that our data do not indicate that *C. picta* reproduces on any other plant but *Malus* spp., and that it is correctly considered a monophagous psyllid species with regard to the host range of immatures. Furthermore, we found DNA of plants in which ‘*Ca.* P. mali’ has been detected in previous studies, indicating that these plant taxa might play a role in *C. picta*-mediated phytoplasma transmission. Thus, our results also provide an important step towards the elucidation of the role of *C. picta* feeding plants in the phytoplasma transmission cycle.

## 5. Conclusions

Our data show, for the first time, that adult *Cacopsylla picta* carry DNA from many different woody and herbaceous plant taxa which do not belong to the host plant of the insect, i.e., *Malus* spp. These findings show that these insects do not stay exclusively on their host plant during their dwell time in the apple orchard. Interestingly, the results of our study point out that herbaceous plants, which *C. picta* has rarely been associated with, are also used for transit and feeding. *C. picta* transmits ‘*Ca*. P. mali’, i.e., the causative agent of apple proliferation disease, and the role of these feeding plants as transmissive or dead-end hosts remains elusive. The dietary composition of *C. picta* seems to be influenced by the fauna at the sampling location. The identification of DNA from the Pinaceae plant family strengthens the hypothesis that members of the genus *Cacopsylla* feed on conifers during hibernation.

## Figures and Tables

**Table 1:** The sampling locations of 95 *C. picta* remigrants (Remi) and emigrants (Emi) from Trentino (TR) and South Tyrol (ST); *n* = number of specimens (in brackets, the number of specimens that were used for the subsequent sequence analysis. Three insects had to be removed from the sample set because no evaluable sequencing results could be obtained from these specimens); Method = sample type which was either beat tray (Beat) or yellow sticky traps (Trap); Date = collection date; Status = migratory status.

Sample Location	Region	Coordinates	Sea Level (m s.l.m.)	*n*	Specimen Code	Type	Date	Status
Bosentino	TR	46°0′ N, 11°13′ E	688	32 (31)	Bosentino 1–31	Beat	4 May 2016	Remi
Eyrs	ST	46°63′ N, 10°65′ E	900	4	Eyrs 1	Trap	21 April 2015	Remi
					Eyrs 2	Trap	29 April 2015	Remi
					Eyrs 3	Beat	21 April 2015	Remi
					Eyrs 4	Beat	3 May 2016	Remi
Kortsch	ST	46°63′ N, 10°75′ E	800	1	Kortsch 1	Beat	29 April 2015	Remi
Latsch	ST	46°37′ N, 10°52′ E	639	2	Latsch 1	Beat	29 April 2015	Remi
					Latsch 2	Beat	11 April 2016	Remi
Mais	ST	46°64′ N, 11°19′ E	700	14 (13)	Mais 1	Beat	30 March 2015	Remi
					Mais 2–3	Beat	17 April 2015	Remi
					Mais 4–9	Beat	10 April 2012	Remi
					Mais 10	Beat	2 April 2013	Remi
					Mais 11	Beat	26 April 2013	Remi
					Mais 12	Beat	9 May 2013	Remi
					Mais 13	Beat	22 May 2013	Remi
Mais	ST	46°64′ N, 11°19′ E	700	24 (23)	Mais 14–21, 26	Beat	26 June 2013	Emi
					Mais 22–24	Beat	20 June 2012	Emi
					Mais 25	Beat	18 June 2013	Emi
					Mais 27–32	Beat	9 July 2013	Emi
					Mais 33–36	Beat	4 July 2013	Emi
Mais Moarhof	ST	46°66′ N, 11°19′ E	600	2	Mais Moarhof 1	Beat	7 May 2013	Remi
					Mais Moarhof 2	Beat	3 May 2013	Remi
Niederlana	ST	46°37′ N, 11°9′ E	310	1	Niederlana 1	Beat	7 May 2015	Remi
Partschins	ST	46°41′ N, 11°4′ E	626	2	Partschins 1	Beat	10 April 2012	Remi
					Partschins 2	Beat	16 April 2014	Remi
Rabland	ST	46°67′ N, 11°06′ E	525	1	Rabland 1	Beat	9 May 2013	Remi
Tarsch	ST	46°36′ N, 10°53′ E	800	1	Tarsch 1	Beat	29 April 2015	Remi
Tartsch	ST	46°68′ N, 10°56′ E	1029	1	Tartsch 1	Beat	22 April 2016	Remi
Tenna/ Bosentino	TR	46°1′ N, 11°16′ E/46°0′ N, 11°13′ E	569/688	3	Tenna/ Bosentino 1–3	Beat	20 April 2015	Remi
Dorf Tirol	ST	46°41′ N, 11°9′ E	594	2	Dorf Tirol 1	Beat	16 April 2013	Remi
					Dorf Tirol 2	Beat	23 April 2013	Remi
Tscherms	ST	46°38′ N, 11°9′ E	292	5	Tscherms 1	Beat	27 March 2012	Remi
					Tscherms 2–5	Beat	03 April 2012	Remi

**Table 2:** Plant genera and families detected in *C. picta* with *trnH* and *rbcLa*; *n* = number of specimens with the determined taxa; (%) = percentage of specimens with determined taxa; *n* reads = number of total sequence reads in order of size; mean ± SD = average number of sequence reads per insect ± standard deviation.

Order	Family	Genus	*n*	(%)	*n* Reads	Mean ± SD	Locus
Rosales	Rosaceae	-	69	75	4506	65 ± 18	*rbcLa*
Rosales	Rosaceae	*Malus*	80	87	307,347	3842 ± 1022	*trnH*
Rosales	Ulmaceae	*Ulmus*	4	4	1801	450 ± 419	*trnH*
Rosales	Cannabaceae	*Humulus*	1	1	159	159	*trnH*
Rosales	Urticaceae	*Urtica*	1	1	1794	1794	*trnH*
Coniferales	Pinaceae	-	17	18	884	52 ± 17	*rbcLa*
Coniferales	Pinaceae	*Picea*	6	7	727	121 ± 61	*trnH*
Coniferales	Pinaceae	*Pinus*	6	7	375	62 ± 28	*trnH*
Coniferales	Pinaceae	*Cedrus*	5	5	6189	1238 ± 807	*trnH*
Coniferales	Cupressaceae	*Juniperus*	1	1	111	111	*trnH*
Fagales	Betulaceae	-	12	13	134	11 ± 2	*rbcLa*
Fagales	Betulaceae	*Betula*	9	10	706	78 ± 23	*trnH*
Fagales	Fagaceae	-	9	10	172	19 ± 5	*rbcLa*
Fagales	Fagaceae	*Quercus*	6	7	156	26 ± 11	*trnH*
Fagales	Fagaceae	*Fagus*	1	1	274	274	*trnH*
Fagales	Juglandaceae	*Juglans*	10	11	12,472	1247 ± 864	*trnH*
Lamiales	Oleaceae	*Fraxinus*	19	21	1983	104 ± 36	*trnH*
Caryophyllales	Polygonaceae	*Rumex*	15	16	5946	396 ± 242	*trnH*
Malpighiales	Salicaceae	*Salix*	10	11	4759	476 ± 273	*trnH*
Malpighiales	Salicaceae	*Populus*	4	4	767	192 ± 96	*trnH*
Cucurbitales	Cucurbitaceae	*Cucurbita*	5	5	965	193 ± 88	*trnH*
Cucurbitales	Cucurbitaceae	*Cucumis*	3	3	415	138 ± 115	*trnH*
Asterales	Asteraceae	*Taraxacum*	4	4	325	81 ± 20	*trnH*
Asterales	Asteraceae	*Lactuca*	2	2	117	58 ± 19	*trnH*
Apiales	Apiaceae	*Daucus*	2	2	1986	993 ± 507	*trnH*
Grimmiales	Grimmiaceae	*Schistidium*	1	1	725	725	*trnH*
Vitales	Vitaceae	*Vitis*	1	1	153	153	*trnH*

**Figure A3:**
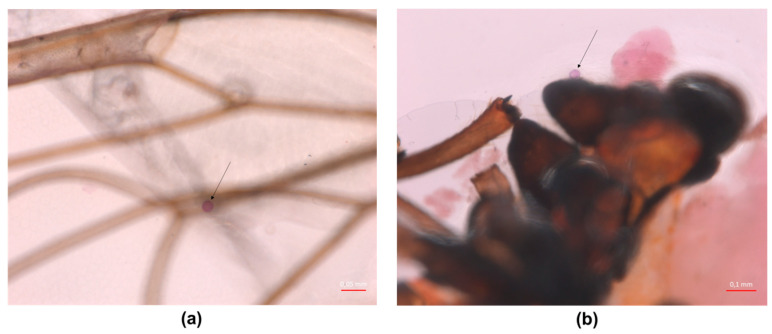
Detection of pollen grains (indicated with a black arrow) on the body surface of *C. picta* (**a**) on a wing; and (**b**) on genal processes.

## Supplementary Materials

The following are available online at https://www.mdpi.com/2075-4450/11/12/835/s1, Table S1: Number of sequence reads for each *C. picta* specimen and plant taxa for the *trnH* locus. Table S2: Number of sequence reads for each *C. picta* specimen and plant taxa for the *rbcLa* locus.

## Appendix A

**Table A1:** Statistical parameters for the total numbers of sequence reads before (raw) and after (clean) quality control for the two loci (*trnH* and *rbcLa*).

Locus	Status	Min	Mean	Max	Sd	Total
*trnH*	raw	22	4464	71,841	9935	424,109
*trnH*	clean	20	4141	69,315	9577	393,442
*rbcLa*	raw	7	813	2754	167	78,026
*rbcLa*	clean	2	144	1143	167	13,780

**Table A2:** Number of pollen grains detected on *C. picta* specimens; *n* = number of pollen grains, nTypes = number of pollen taxa.

Sample Name	*n*	nTypes	Sex
*C. picta* 1	1	1	female
*C. picta* 2	4	4	female
*C. picta* 3	3	3	female
*C. picta* 4	3	1	female
*C. picta* 5	2	2	female
